# Cortical Plasticity during Motor Learning and Recovery after Ischemic Stroke

**DOI:** 10.1155/2011/871296

**Published:** 2011-10-26

**Authors:** Jonas A. Hosp, Andreas R. Luft

**Affiliations:** ^1^Clinical Neurorehabilitation, Department of Neurology, University of Zurich, 8091 Zurich, Switzerland; ^2^Rehabilitation Institute and Technology Center (RITZ), 8008 Zurich, Switzerland; ^3^Division of Brain Injury Outcomes, Department of Neurology, Johns Hopkins University, Baltimore, MD 21231, USA

## Abstract

The motor system has the ability to adapt to environmental constraints and injury to itself. This adaptation is often referred to as a form of plasticity allowing for livelong acquisition of new movements and for recovery after stroke. We are not sure whether learning and recovery work via same or similar neural mechanisms. But, all these processes require widespread changes within the matrix of the brain. Here, basic mechanisms of these adaptations on the level of cortical circuitry and networks are reviewed. We focus on the motor cortices because their role in learning and recovery has been investigated more thoroughly than other brain regions.

## 1. Introduction

From the first steps as a baby to learning the use of a cane in senescence, the human motor system is challenged to acquire novel movement sequences thus enabling a versatile interaction with the environment. Especially at higher age, the integrity of the motor system is threatened. Ischemic brain injury, the major cause of disability in adults [1], affects the motor cortices, their descending pathways, the basal ganglia, or the cerebellum typically leading to a hemisyndrome with motor and sensory deficits affecting arm, leg, and face of one side. After such injury the motor system can reorganize itself to enable partial, sometimes complete recovery of motor function. Apart from acute stroke treatment (e.g., thrombolysis therapy) that intents to prevent the ischemic lesions, neurorehabilitation is the only therapeutic option to reduce disability once infarction is manifest.

Motor learning, recovery after stroke, and neurorehabilitation all depend on the plasticity of neurons and circuits within the motor system. In general, neuroplasticity is defined as the ability of the brain to change its structure and/or function in response to internal and external constraints and goals [2]. This review focuses on three distinct conditions inducing plasticity: (1) skilled *de novo* learning of novel movement sequences in healthy individuals, (2) “spontaneous” (i.e., without any specific training or intervention) cortical reorganization after ischemic injury, and (3) coincidence of “spontaneous” reorganization and relearning of skilled movement sequences with a neurorehabilitative training in the injured brain.

The motor system consists of cortical (primary and secondary motor areas) and extracortical areas (basal ganglia and cerebellum). Furthermore, a close interaction with sensory systems (e.g., the primary somatosensory cortex, S1) is a prerequisite for proper movement execution and motor learning [3, 4]. The observation that movement learning requires protein synthesis in M1 reflecting a plastic storage mechanism [5, 6] highlighted this region as the most likely candidate where motor memories are stored [7]. As its role in learning and recovery has been investigated more thoroughly than other brain regions, this paper will focus on M1. 

The purpose of this paper is to provide a comprehensive overview of plasticity phenomena in the motor cortex during motor skill learning, recovery after injury, and rehabilitation-induced restoration of functional recovery.

## 2. Motor-Learning-Related Cortical Plasticity

### 2.1. Network Plasticity in M1: Learning-Induced Changes in Motor Maps

#### 2.1.1. The Motor Map Illustrating the Somatotopic Organization of M1

Neurons within the primary motor cortex are organized in assemblies that share similar input and output properties to control specific movements over different joints and muscle groups [8]. Therefore an assembly projects to several spinal motoneuron pools [9, 10]. To enable orchestrated multijoint movement sequences, different assemblies are interconnected via horizontal intracortical projections that can spread across several millimeters [9]. Discrete movements and body parts are represented multiple times and are intermingled with representations of related movements or parts forming a complex mosaic pattern. Nevertheless, the primary motor cortex contains a rough somatotopic organization on a larger scale that is highly preserved across species [11], although a fair overlap exists among contiguous representations [12]. This global arrangement can be visualized by direct and transsynaptic electrical stimulation of layer V primary motor neurons using intracortical or epidural microstimulation (ICMS or EMS) [13]. The resulting “motor map” reflects the output pattern of M1. Although the largest body of evidence related to M1 organization on microlevel is derived from studies in nonhuman primates [8, 14], basic features should also be applicable to rodents [14, 15].

#### 2.1.2. Map Changes in Response to Motor Learning

Rats that were trained to retrieve a food pellet show an enlargement in the representation of digits and wrist in the caudal motor cortex [16]. The overall size of the whole forelimb remained unchanged because the enlargement of the distal forelimb occurred at the expense of the proximal forelimb representation. Similarly, squirrel monkeys learning a small object retrieval task have expanded finger representations in the contralateral M1 at the expense of wrist and forearm representations [17]. When digit training is stopped and wrist training started, this expansion is reversed in favor of the wrist representation. Learning-induced enlargement of limb representations was also observed in humans [18]. The contralateral digit representation in M1 as measured by transcranial magnetic stimulation (TMS) was significantly enlarged after learning a finger sequence on the piano.

Taken together, expansion of motor cortex representations during movement training is characterized by: (1) being specific for the trained skill and not induced by motor activity that does not involve learning. In rats, simple lever pressing [16], reaching for an unattainable pellet [19], strength training [20], or reaching without grasping and retrieving [21], did not induce motor map changes. In squirrel monkeys, taking food pellets out of large instead of small wells failed to change M1 somatotopy [22]. (2) The area of enlargement is confined to the cortical area controlling the trained body part. (3) The degree of expansion is correlated with learning success [21]. (4) Finally, map plasticity seems to be essential for successful motor learning. If map plasticity is suppressed, for example, by damaging cholinergic afferents to cortex, learning a reaching task becomes less efficient [23].

In summary, the causal relationship between motor cortex map plasticity and motor learning is highly plausible.

#### 2.1.3. Are Learning-Dependent Map Changes a Substrate for Motor Engrams?

However it cannot be excluded that subtle map changes occur earlier during skill acquisition, a map enlargement measurable by ICMS requires several days and sufficient movement repetitions to develop [19, 24]. In rats, the expansion of forelimb representation emerged between days 7 and 10 at a time when performance had plateaued [19]. Because map changes are present after the skill was successfully acquired, they could be a reflection of the motor engram [7]. But, after training is discontinued, the expansion is quickly reversed [21]: after 8 days of rest, the representation of the trained forelimb assessed by epidural microstimulation reverted to baseline although the motor skill was retained. If map changes are a substrate of this memory, they should actually persist for as long as the skill is remembered. Therefore, transient representational changes may not be reflections of a motor memory trace but may instead indicate the “learning mode” of the system in which storage processes are possible. This mode is reversed once learning has taken place. As an alternative hypothesis, the map enlargement could reflect an intermediate storage of the motor memory trace within M1 that is transferred to different cortical or subcortical brain regions during a later consolidation process. 

In contrast to this hypothesis, increased movement-related M1 activation has been reported using functional magnetic resonance imaging, and this increase was maintained after training ended [25]. But changes in movement-related activation are fundamentally different than alterations in evoked movements in response to cortical stimulation: movement-related activation reflects neuronal populations in control of a movement, and the constituents of these populations change during training. In contrast, results of stimulation mapping depend on the organization of cortical output pathways and on cortical excitability. While the stimulus in measuring movement-related activation is physiological, cortical electrical stimulation is not. Therefore, movement-related activation may be a better surrogate marker for the motor memory trace.

### 2.2. Mechanisms Underlying Map Reorganization and Encoding Motor Memory

#### 2.2.1. Learning-Induced Structural Plasticity within M1

The functional adaptation in M1 that accompanies motor skill learning depends on restructuring of M1 microcircuitry. In rats trained to reach, pyramidal neurons (PMN) in layers II/III and V have enlarged dendritic fields [26, 27]. This enlargement of dendritic surface is accompanied by an increase in the number of synapses per neuron in layer V PMNs suggesting that learning promotes synaptogenesis [19]. In transgenic mice that express yellow fluorescent protein (YFP) in PMNs, learning-induced synaptic remodeling was observed by imaging layer II/III dendritic branches during and after reach training [28]. Formation and elimination of spines, the postsynaptic elements of excitatory synapses in cortex, were documented using two-photon microscopy. Two phases of learning-induced synaptogenesis were distinguished: (1) an early phase of increased spine formation that begins 1 hour after the first training session and lasts up to 4 days (skill acquisition phase), and in which spine density in layer II/III dendritic branches increases and (2) a delayed phase (skill maintenance phase, day 5 to 16) of increased spine elimination, returning spine density to baseline levels. The magnitude of spine formation during the early phase was correlated with learning efficacy. Spines that were formed in this phase became stabilized and were still detectable long after training ended. Overtraining of the same task did not induce further spine turnover, but training a new task did. Interestingly, spines that have been generated during the first task were preserved while training the second task. Altogether, these data suggest that motor learning is associated with rapid but lasting synaptic reorganization. Such structural changes do not occur randomly within the M1 circuitry but are confined to a subset of neurons directly related to a novel motor experience [29] and may represent a footstep of the motor memory trace.

#### 2.2.2. Alterations of Synaptic Weights

Apart from spine and synapse formation, alterations in electrophysiological properties of M1 neurons may contribute to learning-related network reorganization. Likely, long-lasting alterations of synaptic efficacy such as long-term potentiation (LTP) or long-term depression (LTD) are functional correlates of learning-induced plasticity. Both can be induced in M1 *in vitro* [30] and *in vivo* [31]. Rioult-Pedotti and coworkers showed that motor skill learning is associated with LTP-like synaptic plasticity in rats. Acquisition of a reaching task induced a long-lasting increase in synaptic strength in horizontal connections of layer II/III in the M1 forelimb representation contralateral to the trained paw. No changes of synaptic efficacy were detectable in the hindlimb representation [32]. After five days of training, the ability to induce LTP within these connections was partially occluded while LTD increased, suggesting that motor learning expended the capacity of LTP formation [33]. Several weeks after the training ended, layer II/III connections remained strengthened whereas the ability to form LTP and LTD was restored to pretraining levels [34]. 

Similar results were obtained in an *in vivo *animal model (rat) introduced by Monfils and Teskey [35]. In rats learning a reaching task, an enhancement in polysynaptic efficacy within transcallosal efferents to the M1 forelimb representation contralateral to the reaching forelimb was found while task performance improved (day 5–8). Furthermore, repeated high- and low-frequency stimulation induced less synaptic potentiation and more depression in the hemisphere contralateral to the trained forelimb when compared with the ipsilateral hemisphere. In contrast to the *in vitro *studies [33], strengthening of synapses decayed after a few days and vanished at plateau performance (beyond day 8). As the authors hypothesize [35], this difference may be explained by the fact that *in vitro* stimulation affects exclusively layer II/III horizontal pathways whereas stimulation of the corpus callosum *in vivo* produces a generalized unspecific activation of synapses in multiple cortical layers.

### 2.3. Summary

**Figure 1:**
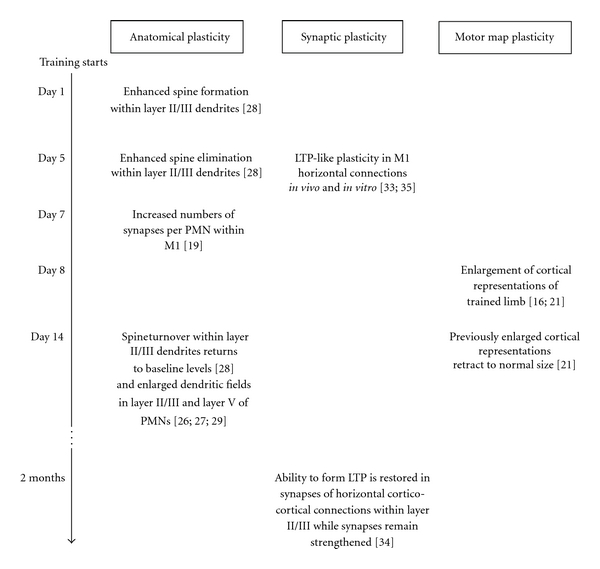
Schematic timescale of plasticity in M1 of rodents at different levels induced by a skilled reaching task. PMN: pyramidal motor neuron; LTP: long-term plasticity.

Based on the present studies performed in rodents, it is possible to extract a rough timescale that reflects several milestones of motor learning-induced plasticity in M1 at different levels (Figure 1). As a limitation of this scheme, it has to be recognized that most of the cited studies focus only on single time points during the learning process, and certain differences in training intensity and training duration between the learning paradigm (reaching task) have to be taken into account. Nevertheless, it allows a conclusion of some basic principles of learning-induced plasticity in M1.

1. Structural modifications and modulation of synaptic weights precede the reorganization of motor maps, suggesting that morphological changes and alterations of connectivity within the M1 microcircuitry form the basis of plastic changes at the network level expressed as an enlargement of motor maps. In line with this hypothesis is the finding that LTP induction produces an expansion of the M1 forelimb representation and of PMN dendritic trees [36]. Vice versa, inducing LTD by low-frequency stimulation of transcallosal projections produces a decrease in dendritic length and spine density in layer III and V of M1 [37].
2. Learning-induced plasticity within M1 follows a biphasic course as an initial “trophic” phase is followed by a period of maturation: after a period of enhanced spine formation some spines are eliminated and spine turnover returns to baseline levels. The ability to form LTP is restored in synapses of horizontal corticocortical connections. Enlarged cortical representations retract to pretraining size.
3. Motor memory may be encoded in primary motor cortex: some of the newly generated synapses that have functional relevance for the learned movement are preserved, and synaptic transmission within horizontal connections remains strengthened. Motor memories may be stored through better connectivity among neurons across M1 to orchestrate the sequential activation of spinal motoneuron pools enabling the execution of movement sequences.

## 3. Cortical Plasticity during Recovery after Ischemic Stroke

### 3.1. Motor Maps Reorganize after Ischemic Stroke and Subsequent Rehabilitation

The reorganization of motor maps after brain lesions was studied in squirrel monkeys [38]. After lesioning approximately 30% of the digit representation in M1 the animals recovered “spontaneously”, that is, without any specific training. After one month, the digit representation in the damaged hemisphere was decreased in size by more than 50%. After 4 months, the reduction was still 25%. This shrinkage was accompanied by an enlargement of the representations of elbow and shoulder. Thus, even small ischemic lesions to M1 induce a profound reorganization of the cortical network in the peri-infarct cortex. 

The shrinkage of the hand representation was prevented by rehabilitative training [39]. This training consisted of restricting the use of the unimpaired hand thereby enforcing the use of the affected hand, a therapy that evolved into the constraint-induced movement therapy (CIMT) that has shown effectiveness in humans [40]. The effect of rehabilitative training depended on its timing [41]. If training was started one month after lesioning, shrinkage of the cortical hand territory occurred despite training. These results indicate the existence of a critical time window during the first weeks after an ischemic stroke in which “spontaneous” reorganization in the M1 network takes place and can be externally influenced by a neurorehabilitative training. But, despite these effects on cortical somatotopy, training did not influence hand function: monkeys that recovered without specific training had similar deficits to trained animals [38]. 

In rat models, training impacted functional recovery as well. Rats trained daily in a reaching task after stroke showed a significantly better functional recovery than untrained animals [42]. Training effects were largest when training was started early [43]. That training improved recovery in rats but not in monkeys likely has methodological reasons (lesion size and testing of motor function). 

In humans with a cortical or subcortical stroke (on average 2 months after stroke) the representation of the abductor digiti minimi muscle (ADM) measured with TMS was smaller in the lesioned hemisphere as compared with the contralateral hemisphere or with healthy controls [44]. After 8–10 weeks of rehabilitative training according to the Bobath approach, the ADM representation enlarged again. The enlargement correlated with the improvement of hand motor function (Canadian Neurological Scale hand score of 0.43 before and 0.9 after therapy).

In summary, ischemic strokes cause reorganization in M1 networks of the peri-infarct cortex and beyond. At least in animal models, this reorganization takes place within the first four weeks after a stroke was induced. Within this dynamic remodeling phase, the network is especially sensitive to therapeutic interventions suggesting the occurrence of synergistic effects on plasticity when training coincides with lesion-induced (spontaneous) reorganization [45].

### 3.2. Structural Plasticity in the Peri-Infarct Cortex (PIC)

Structural changes within the cortical microcircuitry have been examined in the area adjacent to the lesion, the peri-infarct cortex (PIC). This region seems to be particularly important for functional recovery. In humans movement-related activation of PIC in fMRI is correlated with good outcome [46]. 

#### 3.2.1. Dendritic Remodeling and Synaptogenesis in the PIC

In mice, apical dendritic arbors in layer V PMN in PIC close to the ischemic lesion showed extensive remodeling in the form of dendritic tip growth and retraction [47]. Gain and loss of dendritic branches was balanced, and no significant differences of total dendritic length were observed. Interestingly, the degree of dendritic remodeling was smaller with greater distance from the infarct border: there, dendritic arbors were reduced within the first three months after stroke [48]. After photothrombotic stroke in mice, spine density was initially decreased by 38% in the PIC at 24 hours [49]. Subsequent assessments showed an increased spine turnover rate in apical dendrites of layer V PMN that reverted to baseline after 6 weeks. 

In summary, structural changes in dendrites occur in the PIC with their maximum close to the infarct border. In the vicinity of the lesion an initial loss of spine density is followed by increased dendritic remodelling and synaptic turnover. The shrinking of dendritic trees distant to the lesion may be a consequence of a lesion-related reduction in afferent signals. This phenomenon resembles the model of diaschisis [50], describing (dysfunctional) effects of focal brain injuries on remote areas, for example, caused by neuronal deafferentation or redistribution of blood perfusion [50].

#### 3.2.2. Axonal Sprouting in the PIC

The modifications of neural circuitry are not limited to synapses and dendrites. Novel axons are formed as well [51]. In the intact adult brain, axonal sprouting is usually inhibited by three different classes of inhibitory proteins [52]: extracellular matrix proteins forming perineuronal networks (e.g., tenascin and chondroitin sulfate proteoglycanes), myelin-associated proteins (e.g., NogoA and myelin-associated glycoprotein), and developmentally associated growth-cone inhibitory proteins (e.g., molecules of the ephrin and semaphorin classes). 

**Figure 2:**
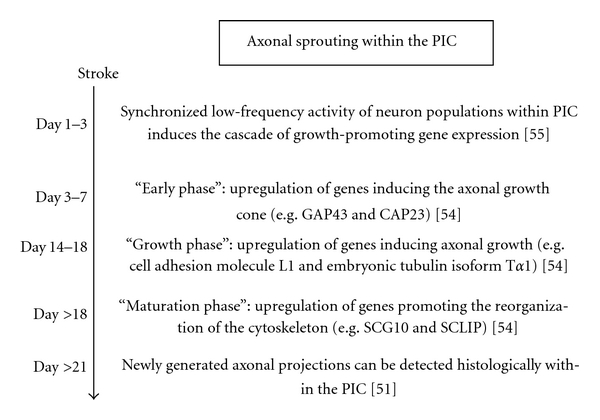
Timescale illustrating the milestones of axonal sprouting within the PIC after a photothrombotic cortical stroke in rats.

Around the infarct core apoptotic cell death and gliosis dominate [53]. Within this region of the gliotic scar, both growth-promoting and inhibitory genes are overexpressed [54]. At greater distances from the infarct, in the area surrounding the scar, inhibitory perineuronal networks degrade due to inflammatory processes and free radical formation, thereby facilitating axonal sprouting [45]. In this area, growth-promoting genes are upregulated, and inhibitory genes are downregulated [54]. Thus, the PIC can be subdivided into a gliotic scar region surrounded by a growth-permissive zone [45, 54, 55]. In this growth-permissive zone a defined cascade of genes becomes induced after stroke enabling the formation of new axonal projections (Figure 2). This pattern of gene expression seems to be specific for reorganization after stroke and differs from axonal growth during development or recovery from peripheral nerve injury. 

The temporal sequence of these events depends on age [56]: in aged rats, growth-promoting genes are expressed later, and growth-inhibiting genes are expressed earlier than in younger animals. Such differences in gene expression and patterns of remodeling may be the reason for the worse outcomes of elderly stroke survivors.

#### 3.2.3. Does Synaptic Plasticity Occur in the PIC?

If and to what extent long-term synaptic plasticity like LTP and LTD contributes to network reorganization after stroke is still unresolved. Hagemann and colleagues reported enhanced LTP in the perilesional zone around a photothrombotic lesion to primary somatosensory cortex (S1) [57]. Neurons in the PIC are more excitable after a stroke because NMDA-receptor expression is upregulated [58] and GABA-A-receptors are downregulated [59]. Reducing inhibition by blocking GABA-A-receptors is usually a prerequisite for the induction of LTP *in vitro*. Therefore increased cortical excitability might be a plausible explanation for facilitated LTP in the PIC.

### 3.3. Poststroke Plasticity Beyond the Peri-Infarct Cortex (PIC)

Beyond the PIC other brain regions are involved in poststroke reorganization. Three months after lesioning the M1 hand representation (>50% destruction) of squirrel monkeys, ICMS revealed an enlargement of the hand representation of the ventral premotor cortex (PMv); the magnitude of enlargement was proportional to the shrinkage of the M1 hand area [60]. In contrast, smaller infarctions (<50% destruction of the hand representation) that caused only mild deficits in the affected limb lead to shrinkage of the PMv hand representation [61]. The PMv is intensely interconnected with M1. Functionally, it is thought to contribute to sensory guidance of movements as well as movement preparation [8]. The PMv is involved in recovery processes after brain lesions in monkeys [62] and stroke patients [63]. Movement-related activation in PMv is increased after rehabilitative training that improves arm function [64]. These findings suggest that PMv contributes to recovery if M1 reorganization is not possible—as it is the case after large lesions.

Postlesional reorganization occurs not only within PMv but also in its connections with other cortical regions. Neuroanatomical tracing five months after stroke showed new projections between PMv and S1 [65]. These connections may compensate for interrupted S1-to-M1 projections. New PMv-S1 fibers may improve the functional coupling between sensory and motor systems.

Larger strokes comprising the distal forelimb representation in M1 and the premotor cortex in squirrel monkeys resulted in severe motor deficits. These deficits recovered only partially [66]. ICMS revealed an expansion of the hand representation in the supplementary motor cortex (SMA). The amount of expansion was proportional to lesion size and was positively correlated with postlesional recovery. The SMA contributes to the control of posture, to initiation and execution of limb movements as well as to the synchronization between M1 regions in both hemispheres [8]. Bilateral activation of SMA in an fMRI study was associated with good recovery after stroke [67].

In summary, these studies show that secondary motor areas are involved in remodeling processes initiated by lesions to M1. Although this remodeling shows a correlation to functional recovery, the question of a causal relationship remains unanswered. 

The changes described above all occur in the ipsilesional hemisphere but the contralesional hemisphere undergoes reorganization as well. In rats, dendrites of PMN expand in layer V of the contralesional M1. This expansion is followed by dendritic pruning [68]. The growing of dendrites is accompanied by synaptogenesis [69]. These structural modifications may be the consequence of compensatory overuse of the unimpaired limb [70]. In humans, fMRI or PET studies revealed an activation of the contralesional hemisphere predominantly in the first days to weeks after a stroke [71, 72]. However, in individuals with good recovery, brain activation during paretic limb movement shifts towards the ipsilesional hemisphere. Persistence of contralesional activation is related to poor recovery [72, 73]. But, bilateral arm training evokes contralesional premotor activation which is associated with a good therapy response [64]. Therefore, the functional role of the unlesioned hemisphere after stroke and its contribution to recovery are still unresolved. It may be that temporary involvement of the contralesional hemisphere is necessary during an early stage of recovery.

### 3.4. Implications for Clinical Practice

After an ischemic stroke affecting M1, profound reorganization occurs around the infarct as well as in remote areas of cortex within both hemispheres. Such reorganization is accompanied by partial or complete recovery of motor function. Structural changes like axonal sprouting, dendritic remodeling, and synapse formation occur—at least in animal models—during the first weeks after infarction. During this critical time window, reorganization processes can be influenced and optimized by training. In consequence, spontaneously occurring plasticity should be exploited by starting rehabilitation as early as possible. But also late after stroke (>6 months), reorganization is still possible, and functional recovery occurs in almost all individuals in response to intense training [64, 74]. Certain areas of cortex like PMv and SMA are able to remodel and thereby compensate for lesions in M1. It remains to be elucidated which patterns of reorganization occur when other areas of the brain or all sensory and motor cortices are damaged.

## 4. Do *De Novo* Learning and Relearning Movement Sequences during Rehabilitation after Stroke Depend on Similar Mechanisms?

As discussed above, *de novo *motor-learning-induced alterations on the level of microcircuitry (dendrites and synapses) and network reorganization (motor maps) in the intact M1 are well examined, emphasizing that this structure plays a plausible role for the storage of newly acquired motor memories. For the relearning of movement sequences after an ischemic stroke, corresponding data are lacking. Currently, one assumes that *de novo* motor learning requires more or less similar plastic modifications like relearning during rehabilitation [75]. However, obvious differences exist between both conditions: given that M1 is a key structure for the storage motor engrams, it is poorly understood where and how novel motor memory is stored in the injured brain, especially when M1 is damaged or underwent a lesion-induced reorganization. Furthermore, relearning movement sequences may be hindered by an interference of “residual” elements of previously stored memory traces [76] or dysfunctional spontaneous reorganization patterns [77]. Therefore, it seems premature to consider *de novo* motor learning as an appropriate model for rehabilitation-induced recovery. Further studies investigating rehabilitation-induced plasticity on the cellular and network level are required to decide if knowledge from motor learning can be transferred to optimize neurorehabilitation strategies or if completely different concepts have to be developed.
